# COVID19-CT-dataset: an open-access chest CT image repository of 1000+ patients with confirmed COVID-19 diagnosis

**DOI:** 10.1186/s13104-021-05592-x

**Published:** 2021-05-12

**Authors:** Shokouh Shakouri, Mohammad Amin Bakhshali, Parvaneh Layegh, Behzad Kiani, Farid Masoumi, Saeedeh Ataei Nakhaei, Sayyed Mostafa Mostafavi

**Affiliations:** 1 Department of Medical Informatics, School of Medicine, Mashhad University of Medical Sciences, Mashhad, Iran; 2 Department of Radiology, Faculty of Medicine, Imam Reza Hospital, Mashhad University of Medical Sciences, Mashhad, Iran; 3 Nuclear Medicine Research Center, School of Medicine, Mashhad University of Medical Sciences, Mashhad, Iran

**Keywords:** Coronavirus, COVID-19, Computed tomography, Chest CT image, Lung infection, Diagnosis, Artificial intelligence, Deep learning, Clinical imaging, Radiology

## Abstract

**Objectives:**

The ongoing Coronavirus disease 2019 (COVID-19) pandemic has drastically impacted the global health and economy. Computed tomography (CT) is the prime imaging modality for diagnosis of lung infections in COVID-19 patients. Data-driven and Artificial intelligence (AI)-powered solutions for automatic processing of CT images predominantly rely on large-scale, heterogeneous datasets. Owing to privacy and data availability issues, open-access and publicly available COVID-19 CT datasets are difficult to obtain, thus limiting the development of AI-enabled automatic diagnostic solutions. To tackle this problem, large CT image datasets encompassing diverse patterns of lung infections are in high demand.

**Data description:**

In the present study, we provide an open-source repository containing 1000+ CT images of COVID-19 lung infections established by a team of board-certified radiologists. CT images were acquired from two main general university hospitals in Mashhad, Iran from March 2020 until January 2021. COVID-19 infections were ratified with matching tests including Reverse transcription polymerase chain reaction (RT-PCR) and accompanying clinical symptoms. All data are 16-bit grayscale images composed of 512 × 512 pixels and are stored in DICOM standard. Patient privacy is preserved by removing all patient-specific information from image headers. Subsequently, all images corresponding to each patient are compressed and stored in RAR format.

## Objective

Coronavirus disease 2019 (COVID-19) is an infectious, highly contagious disease with major global health implications. As of January 31, 2021, there have been 103 million confirmed infections worldwide, claiming over 2.2 million lives [1, 2]. A major hurdle in the management and control of COVID-19 is availability of timely disease screening and monitoring tests.

Computed tomography (CT) scans are routinely used in clinical practice for diagnosis, screening and management of COVID-19 worldwide. The heavy number of required scans keeps radiologists highly occupied and leaves them with limited time, which hinders the delivery of timely CT reports. Besides, there is limited access to well-trained radiologists with adequate COVID-19 imaging expertise in many underdeveloped rural regions. Collectively, these call for data-driven Artificial intelligence (AI)-powered solutions for automatic detection and quantification of COVID-19 infections.

To date, there have been numerous studies that have attempted to deploy AI-based approaches, such as deep convolutional neural network (CNN) models, for automatic detection and quantification of COVID-19 from CT images [3–5]. The key to success of these models is the deployment of rich datasets that encapsulate diverse patterns of lung infections. However, owing to privacy and data collection issues, CT images used in these studies are limited in size and not publicly available. This significantly impacts the development of new AI-powered solutions for more advanced diagnosis and quantification of COVID-19 infections.

Herein, we present an open-access repository of 1000 + CT images obtained since the onset of COVID-19 in March 2020, at least one order of magnitude larger than the current available datasets [6–8]. Given the diverse patterns of infection covered in this rich dataset, this can serve as the starting point for more comprehensive data-driven models. Moreover, this can also be used as an educational resource for under-trained radiologists in less developed areas around the globe.

## Data description

This dataset consists of unenhanced chest CTs from 1000+ patients with confirmed COVID-19 infections. The age distribution of patients who underwent CT imaging was 47.18 ± 16.32 (mean ± standard deviation) years and age range was between 6 and 89 years. Gender distribution was 60.9% male and 39.1% female. The most prevalent self-reported coexisting conditions among patients included hypertension or coronary heart disease, diabetes, and interstitial pneumonia or emphysema (in that order). Images were obtained in the March 2020–January 2021 period, and were acquired at the point of care in an inpatient setting from patients with positive Reverse Transcription Polymerase Chain Reaction (RT-PCR) tests for COVID-19, accompanied by supporting clinical symptoms. All scans were performed with the patient in the supine position during end-inspiration. The scanning range was from the apex to lung base. CT exams were performed with a NeuViz 16-slices CT scanner machine (Neusoft medical systems) without intravenous contrast under “Helical” mode. All images are in DICOM format and consist of 16-bit grayscale images composed of 512 × 512 pixels. Slice thickness values were determined by the operator in accordance with clinical examination requirements: 1.5 or 3 mm. Patient privacy is preserved by removing all patient-specific information from image headers. Subsequently, all images corresponding to each patient are compressed and stored in RAR format. Table 1 provides an overview of the dataset.

**Table 1:** Overview of dataset

Label	Name of data set	File types (file extension)	Data repository and identifier (DOI or accession number)
Data set 1	Covid19-CT-dataset	RAR files (.rar)	Harvard Dataverse (https://doi.org/10.7910/DVN/6ACUZJ) [10]

All CT images were visually examined by two board-certified radiologists for the presence of COVID-19 infections. In case of a disagreement between the first two radiologists, a third more experienced radiologist rendered the final decision. CT images were identified to have a broad mixture of COVID-specific patterns of lung infections including: (i) presence of ground-glass opacities, mixed ground-glass opacities, or consolidation; (ii) presence of air bronchogram, interlobular septal thickening, or cavitation; (iii) different number of lobes affected by ground-glass or consolidative opacities; (iv) presence of fibrotic lesions; (v) presence of centri-lobular nodules; (vi) presence of a pleural effusion; (vii) presence of thoracic lymphadenopathy; (viii) presence of underlying lung disease such as tuberculosis, emphysema, or interstitial lung disease; and (ix) different distribution patterns of opacities including peripheral, central, bilateral, focal, multi-lobar and diffuse. Ground-glass opacification was defined as “hazy increased lung attenuation with preservation of bronchial and vascular margins” and consolidation was defined as “opacification with obscuration of margins of vessels and airway walls” [9].

## Limitations

- A small number of images contain some form of background noise such as patient bed and/or some form of motion artifacts.
- Images were taken from only two general hospitals in Mashhad, Iran, and represent a predominantly Iranian population.

## Data Availability

The data described in this data note can be freely and openly accessed on Harvard dataverse under (https://doi.org/10.7910/DVN/6ACUZJ) [10]. Please see Table 1 for details and link to the data.

## Abbreviations

COVID-19: Coronavirus disease 2019
CT: Computed tomography
AI: Artificial intelligence
RT-PCR: Reverse transcription polymerase chain reaction
DICOM: Digital imaging and communications in medicine
RAR: Roshal archive
CNN: Convolutional neural networks
